# Navigating pregnancy and early motherhood in prison: a thematic analysis of mothers’ experiences

**DOI:** 10.1186/s40352-022-00196-4

**Published:** 2022-10-29

**Authors:** Diksha Sapkota, Susan Dennison, Jyai Allen, Jenny Gamble, Corrie Williams, Nomxolisi Malope-Rwodzi, Laura Baar, Janet Ransley, Tara Renae McGee

**Affiliations:** 1 Griffith Criminology Institute, Griffith University Mount Gravatt Campus, 176 Messines Ridge Road, Mount Gravatt, Qld Australia; 2 Transforming Maternity Care Collaborative, School of Nursing and Midwifery, Griffith University, Nathan, Australia; 3 Coventry University, Priory St Coventry, UK

**Keywords:** Correctional facilities, Focus groups, Mothers, Pregnancy, Qualitative, Young child

## Abstract

**Background:**

Maternal imprisonment negatively impacts mothers and their children and is likely to have lifelong and intergenerational sequelae. In many jurisdictions nationally and internationally, young children (usually those less than 5 years) can reside with their mothers in prison. However, there is considerable debate regarding the impact of prison environments on incarcerated mothers and their children who are born, and/or raised in prison. Research to date on the pregnancy and mothering experiences of imprisoned mothers and their preferences for care arrangements for their babies and young children is limited.

**Methods:**

This study was part of the Transforming Corrections to Transform Lives project, in which workshops were conducted with imprisoned mothers to understand their needs while in custody and post-release, and the kind of supports and system changes that are required to meet those needs. Incarcerated mothers (*n* = 75) participated in seven workshops conducted across four Queensland prisons. Themes were generated through reflexive thematic analysis.

**Results:**

Three themes characterised mothers’ experiences of being pregnant and undertaking a mothering role of a young child while in prison. First, for most mothers, imprisonment adds vulnerability and isolation during pregnancy and childbirth. Second, although mothers felt that residing together with their children in prison motivated them to change for a better future, they were concerned about the potential negative impact of the prison environment on the child’s development. Lastly, most mothers voiced losing autonomy and agency to practice motherhood independently within custodial settings. Mothers expressed a need for the correctional system to be adapted, so it is better equipped to address the unique and additional needs of mothers with young children.

**Conclusion:**

Mothers’ experiences indicated that the correctional system and policies, which were predominantly designed for men, do not adequately address the varied and complex needs of pregnant women, mothers, and their young children. Imprisonment of pregnant women and mothers with young children should be the last resort, and they should be provided with holistic, individually tailored support, most preferably in community settings, to address their multiple intersecting needs.

**Supplementary Information:**

The online version contains supplementary material available at 10.1186/s40352-022-00196-4.

## Background

Globally, female prisoners account for 6.9% of the total prison population and since 2000, female prisoners have increased by 53% (Walmsley, 2017). Most notably, many of these women are mothers — more than 80% have at least one child and 30% have children under five years of age (Glaze & Maruschak, 2010; Lobo & Howard, 2021; Shlafer et al., 2019). Consistent with global figures, the number of women in prison in Australia has increased by 62% (3,292) compared to 2,030 in 2011, while Indigenous women make up 30% of the female prison population (Australian Bureau of Statistics [ABS], 2021). Though research on impact of maternal incarceration and mothering in prison is robust (e.g., Casey-Acevedo et al., 2004; Dowell et al., 2019; Goshin et al., 2014; Nuytiens & Jehaes, 2022), imprisoned mothers’ voices are rarely considered in such research. Mothers’ perspectives about the effectiveness of prison-based programs, including parenting programs, prison nursery programs, or health programs have been investigated (Bard et al., 2016; Kennedy et al., 2020;; Walker et al., 2014). However, little is known about how mothers feel about mothering a young child (from birth to five years) while imprisoned and how the prison system influences their journey of motherhood.

### Pregnant, birthing, and postpartum in prison

Pregnancy and motherhood can be positive turning points for some incarcerated women as they strive to be the best mothers they can be and achieve desistance from crime (Schinkel, 2019). Available estimates indicate that globally 5 to 10% of women in prison are pregnant (Baldwin et al., 2020), while in Australia, in 2017, 1.8% of women in prison were pregnant at the time of entering prison (ABS, 2021; Australian Institute of Health and Welfare [AIHW], 2019). Studies have shown that women, currently or formerly incarcerated, have delayed entry to antenatal care and are less likely than other women to receive the recommended antenatal care (Ramirez et al., 2020; Walker et al., 2014). Early and routine prenatal care during the imprisonment of pregnant women has shown to have positive effects on babies’ birth weight and gestational age at birth (Baker, 2019). However, late and insufficient care leads to poor perinatal outcomes (e.g., pre-term birth, low birth weight, mental illness, and increased hospital admissions and mortality; Baldwin et al., 2020; Walker et al., 2014; Dowell et al., 2018; Mukherjee et al., 2014).

Despite what is known about the increased risks for adverse pregnancy-related outcomes due to incarceration, little data is available on what imprisoned women need to optimise their pregnancy outcomes. Several programs have been implemented globally to improve pregnancy and birth experiences and support the unique mothering needs of incarcerated women (Paynter et al., 2020; Tremblay & Sutherland, 2017). However, most of the existing programs do not extend beyond the postnatal period, have not been rigorously evaluated (Bard et al., 2016), and have not given much consideration to women’s experiences and their preferences during pregnancy and childbirth in prison (Baldwin et al., 2020; Fritz & Whiteacre, 2016). In addition, little is known about what works to improve incarcerated mothers’ health and wellbeing during pregnancy, birth, and postpartum, with scholars concluding that available programs are either insufficient or ineffective in addressing the needs of pregnant and postpartum women (Baldwin et al., 2020; Bard et al., 2016; Paynter et al., 2020). Examining subjective experiences of imprisoned mothers is critical to broaden our understanding on how they can be better supported to ensure their positive pregnancy and birthing experiences.

### Mothering children while in prison

In many countries, including Australia, the rapid rise in female incarceration in recent decades has meant an unprecedented increase in the number of children affected by maternal incarceration (ABS, 2021; Walmsley 2017). An Australian-based study reported that nearly two-thirds of females in prisons have at least one dependent child (Lobo & Howard, 2021) and another study from the United States (US) indicates that nearly a quarter of children with a mother in prison are under five years of age (Shlafer et al., 2019). Compared to paternal incarceration, maternal incarceration may be more consequential for children because it brings more instability in a child’s life, including changes in the primary caregiver, moving houses, or changes in schools or friends. When a father goes to prison, in more than 80% of cases, children will be taken care of by their mothers in their own home (Dennison et al., 2013). However, when a mother goes to prison, children are mostly cared for by grandparents, particularly grandmothers (around 50% of cases), followed by extended family members or friends and state-care in a different location (11%; Glaze & Maruschak 2010).

Children often suffer collateral and damaging consequences of maternal incarceration that can impact their health and wellbeing, behaviour, and educational prospects (Aiello & McCorkel, 2018; Minson, 2019; Poehlmann-Tynan & Turney, 2021). Although the detrimental impacts of parental incarceration on children’s development is well documented, there is a lack of consensus regarding the age-graded effects of maternal incarceration (Poehlmann‐Tynan & Turney, 2021). Most of the research has focused on incarceration of either parent and been conducted with adolescents or included children from a wide age range (from birth to 18 years; Murray & Farrington, 2008; Poehlmann‐Tynan & Turney, 2021). But infants and young children have different needs and face different challenges compared to older children, thus, need differential support. For example, infants and young children rely on their parents, particularly mothers, for regulating emotions and developing healthy attachments (Poehlmann, 2005). Newborn babies and infants who were separated from their mothers experienced a disruption in their attachment and had elevated internalising and externalising problems, compared to those who were not separated (Fritz & Whiteacre, 2016; Poehlmann‐Tynan & Turney, 2021; Poehlmann 2005). Analyses of a linked administrative dataset from Australia revealed that children exposed to maternal imprisonment had higher infant mortality rates, increased risk of developmental vulnerabilities, and were twice as likely to encounter child protection services by the age of two, compared to children of mothers with no involvement in the correctional system (Dowell et al., 2018, 2019).

During the first three to five years of a child’s life, the foundations for learning, health, and behaviour throughout life are laid down. Children’s early life exposures and social environment, including mother-child interactions, significantly predict multiple aspects of their development in later life (Britto et al., 2017). Separating young children from their mother, particularly during the first five years of life, may have long-term emotional damage, because of potential disruptions to their healthy early brain development and reductions in their ability to form primary attachments and develop stable relationships (Britto et al., 2017; Powell et al., 2017). Exposing young children to nurturing talk and interactions and preventing emotional trauma can contribute to improving their cognitive and behavioural skills (Britto et al., 2017; Poehlmann-Tynan & Turney, 2021).

To mitigate the negative effects of maternal-child separation due to incarceration and promote parenting skills, correctional agencies, nationally and internationally, have implemented several prison-based programs targeting mothers with young children. For example, female correctional centres in some countries, including Australia, the United States of America, and the United Kingdom, have allocated units that allow mothers and their young children to remain together in prison; although, there are wide variations across countries in children’s permitted length of stay in prison and the facilities available (e.g., Paynter et al., 2020; Shlonsky et al., 2016; Thomson et al., 2022). Keeping a young child with their mother in prison has been shown to have some positive impacts for both mothers and children, such as improved mother-child relationships, mental health, quality of life, and for mothers, improved rehabilitation and reduced reoffending (Dolan et al., 2019; Fritz & Whiteacre, 2016; Goshin et al., 2014; Paynter et al., 2020; Tuxhorn, 2021). Despite these benefits, there are mixed views regarding impact of the prison environment on growth and development of young children (Dolan et al., 2019; Kennedy et al., 2020; Nuytiens & Jehaes, 2022), as children’s home environment significantly affects their overall development (Yang et al., 2021).

Mothers of young children are given access to parenting programs, which are increasingly being advocated due to their perceived ability to improve parenting skills, strengthen family relationships, reduce the negative impacts of maternal imprisonment on children, and reduce re-offending (Goshin et al., 2014; Tremblay & Sutherland, 2017). However, there is a significant heterogeneity in program design and delivery and only few studies have asked mothers about their parenting education needs (Lovell et al., 2022; Tremblay & Sutherland, 2017).

Parent-child contacts are encouraged as evidence suggests that regular contact between imprisoned mothers and their children, particularly in-person visitation, can prevent the deleterious impacts of separation for both mothers and children, including improvement in their emotional health, relationship quality, and bonding, and better adjustment to maternal incarceration among children (Kennedy et al., 2020; Poehlmann, 2005; Schubert et al., 2016; Haverkate & Wright, 2020; Flynn et al., 2021). However, scholars assert that the benefits derived from visitation programs greatly rely on the quality of the mother-child relationship before incarceration and the relationship between the mother and the child’s current caregivers (Kennedy et al., 2020; Poehlmann-Tynan & Turney, 2021). In addition, because there are fewer correctional facilities for women compared to men, mothers are often housed farther away from their children (Productivity Commission, 2021) and prison visits often get impacted by the cost and logistics associated with travel to facilities (Kennedy et al., 2020; Schubert et al., 2016). In sharp contrast, some studies have reported negative impacts of communication with their children, such as increased rule violations among mothers after writing letters to their children (Benning & Lahm, 2016) or behavioural outbursts among women who receive visits from their children (Casey-Acevedo et al., 2004).

### The current study

As discussed, research on maternal incarceration is emerging but, in most cases, studies have investigated the impacts of prison-based programs on improving outcomes of incarcerated mothers and their children (Shlonsky et al., 2016) or the parenting education needs of mothers (Lovell et al., 2022). Most studies are conducted in a few countries, predominantly the United States and the United Kingdom, and have several methodological limitations, such as non-standardised assessments, lack of comparison groups, and the absence of long-term follow-ups (Dodson et al., 2019; Shlonsky et al., 2016). Voices of imprisoned mothers are almost non-existent in this scholarly arena (Nuytiens & Jehaes, 2022). Where studies have been conducted, women are mostly asked to provide retrospective accounts of their perspectives towards some prison-based programs, particularly prison nursery or parenting programs (Baldwin et al., 2020; Lovell et al., 2022). There has been less emphasis on what mothers themselves believe would be helpful for them to undertake their maternal responsibilities for a young child while in prison. It is not clear how mothers perceive the co-placement of their younger children with them in prison or how they feel that the services presently being offered meet their needs.

Imprisoned mothers with young children are a priority population given their vulnerability and unique needs (e.g., belong to impoverished and marginalised communities, have childhood or adult experiences of abuse and trauma, experience mental illness and drug dependency, and face challenges in reuniting with children and returning to the community). Much of the existing research on maternal incarceration has focused on mothers’ reproductive healthcare needs or parenting or how mothers construct and hold on their identity as a mother while in prison (Baldwin, 2017; Bard et al., 2016; Lovell et al., 2022). Despite evidence supporting the differential impacts of maternal incarceration depending on the child’s age and stage of development (Powell et al., 2017), most studies have included mothers of dependent children but none of them have explored the unique experiences associated with mothering young children while in prison. Undoubtedly, for most mothers, separation from their young children is the hardest aspect of imprisonment (Powell et al., 2017; Walker et al., 2021), yet much scholarly work has explored the impacts of maternal incarceration on children, rather than the impacts of separation upon mothers themselves (Aiello & McCorkel, 2018; Dowell et al., 2019; Murray & Farrington, 2008) or the difficulty in making choices around whether to have a child placed with them in prison. There is mixed evidence around means and frequency of contact that mothers prefer for communicating with their children (Haverkate & Wright, 2020; Flynn et al., 2021; Benning & Lahm, 2016; Casey-Acevedo et al., 2004).

There is a need to broaden our knowledge by illuminating the perspectives of imprisoned mothers as they navigate pregnancy/childbirth, mothering roles, and connections with their young children. Such research is vital to developing holistic, individually tailored supports that work towards improving positive outcomes (e.g., relationship quality, maternal–child bonding, and emotional health) for mothers in prison and their children. This study is valuable for understanding the needs of mothers of young children by empowering them to share their experiences and concerns. The following research questions guided this study:

1. What are the experiences of mothers regarding pregnancy and mothering a young child (from birth to five years) while in prison?
2. What personal, interpersonal, and systemic challenges do mothers in prison face while rearing their young child/ren, whether the child is in prison with them or outside?

## Methods

This research sits within Phase One of the Transforming Corrections to Transform Lives (TCTL) project, aimed at co-creating a model of service delivery to provide holistic care and support to imprisoned mothers and their children.

### The research context

As of July 2021, there were 3,292 women in Australian prisons and out of them, 933 were imprisoned in the five women’s correctional centres across Queensland (ABS, 2021)^1^. All women’s correctional centres in Queensland have provisions for a small number of children to reside with their mothers in mother-baby units, whereas these facilities are less common in other jurisdictions. In 2017, there were 69 children living with their mothers across all Australian prisons, with 34 of these children residing in prisons in Queensland (Anti Discrimination Commission Queensland [ADCQ], 2019). Children of imprisoned women in Queensland can stay with their mothers in prison until they are school-aged (approximately 5 years old), if they meet the specific requirements set by correctional centres (Shlonsky et al., 2016).

### Sampling and recruitment strategy

Purposive sampling was used to recruit mothers in prison, regardless of whether their children were with them in prison or in the community. Research flyers were placed in the prisons and interested women who self-identified themselves as mothers informed correctional staff about their interest to participate in the workshops. Correctional staff ensured that women with serious psychiatric illnesses who were currently symptomatic, those unable to speak English, or exhibiting violent behaviours did not participate in the workshops.

### Characteristics of study participants

A total of 75 imprisoned mothers participated in the workshops. The mothers were aged between 19 and 55 years; the mean age was 33.4 years (*SD* = 8.4). Of these women, 28% (*n* = 21) identified as Aboriginal and/or Torres Strait Islander, which is slightly lower than the proportion of 38% in the national female prison population (ABS, 2021). On average, mothers in prison had two children, although this ranged from one to 11. Altogether, the mothers had 221 children, ranging from infancy to adulthood. Of the 221 children, 55 (25%) were under 5 years old. The number of children residing with their mother in prison was not recorded. At the time of the workshops, mothers reported that they had currently been in prison for less than a month to over nine years. However, excluding an outlier, the average current time in prison was just over seven months (*M* = 7.46, *SD* = 8.97). There were participants from both low and high security prisons. In terms of location where data collection was conducted, 42.7% (*n* = 32) of women were in Northern Queensland, while the remaining 57.3% (*n* = 43) were in South-East Queensland.

### Data collection methods and instruments

In December 2020, seven workshops were conducted in a neutral setting within the prison, without the presence of correctional officers. Workshop sizes ranged between eight and 20 participants. In each workshop, participants were divided into smaller groups of three to five participants. The lead researcher introduced the research project at the start of the workshops, explained the informed consent process, observed discussion, and facilitated discussions when required. The members of the research team facilitated small group discussions; there were 19 small group discussions in total.

The facilitators used semi-structured questions to ask participants about their needs, whether those needs were being met by the prison system, and what help or support services they need, both in prison and post-release. For example, women were asked, “What do you think women, in general, need when they are in prison?” “More specifically, what are the needs of imprisoned mothers and how do you think we can better support them?” During these discussions, women shared their experiences of pregnancy, birthing, and mothering young children, which are described in this paper. The facilitators encouraged participants’ interactions to explore and challenge ideas, and guided discussion back to the topic if it deviated. Each group discussion lasted 60 to 90 min, with 28 h of audio-recorded discussions.

### Data processing and analysis

All group discussions were transcribed using a professional transcription service and stored in a secure computer drive that was only accessible to the research team. Microsoft Excel was used for data management and coding, while Microsoft Word was used to thematically organise quotes. The data were analysed using reflexive thematic analysis which included six phases as recommended by Braun & Clarke 2019. This approach was chosen as it allows flexibility, creativity, and active engagement of researchers, who can use their knowledge in drawing meanings from the data (Braun & Clarke, 2019). This first phase of familiarisation with the data commenced whilst conducting workshops followed by listening to the recordings and reading and re-reading the transcripts. Three researchers independently listened to transcripts from one randomly selected group interview and did line-by-line coding. Through peer debriefing, codes were revised, and discussion continued until all coders agreed on a codebook, which included a list of agreed upon codes, their descriptions, and illustrative quotes. The remaining eighteen audio files were divided among four researchers for coding. Involvement of multiple coders allowed a richer and nuanced understanding of the data. Each researcher took notes during and after the workshop to record their thoughts, ideas, experiences, and observations of the data collection process. Reflexive team discussions typically occurred the day after each workshop regarding the research process and findings. Transcripts and post-workshop notes were reviewed in conjunction with transcripts to ensure codes generated accurately represented the findings of the workshops. The codebook was updated whenever new codes emerged. Themes were generated and refined after several iterative cycles of analysis. The co-authors reviewed, reflected, and discussed how themes were organised and how information was coded.

### Reliability and validity of findings

Reflexive discussions, led by the project lead, explored alternative interpretations of the data, and ensured themes directly reflected the data. Cross-checking emergent themes allowed for credibility-checks. Involvement of multiple coders during the codebook development ensured inter-coder agreement and reliability, and made the coding process a collaborative and reflexive one, contributing to the richer understanding of the data (Roberts et al., 2019; Braun & Clarke, 2019). The shared codebook was regularly updated as new codes emerged, which allowed all evolving ideas to be captured.

### Ethical issues

Ethical approval was provided by the Griffith University Human Research Ethics Committee and permission granted to conduct workshops from the relevant correctional centres. To protect against unnecessary emotional distress, the questions asked during the workshops did not include personal questions about the women’s life histories. However, because it was possible that some mothers might become distressed while discussing their experiences and concerns about their children, participants were provided with the contact details of support services (both in prison and via telephone helplines) and were encouraged to access these services if needed. Personal identifying information was not collected from the participants; however, the participants’ identities were known to other women participating in the workshops. Participants were asked not to reveal the identities of people during group discussions (GD) or disclose information outside of the workshops and were advised not to disclose information that would make them uncomfortable. Participants were informed that they could withdraw from the study without providing reasons at any time up to the conclusion of the workshop. They were provided with a certificate of participation to acknowledge their contributions to the project. To ensure the confidentiality and anonymity of participants and settings, each centre and group discussion were assigned a number and quotations are presented in italics and identified by site and group discussion number (e.g., Centre1, GD1). Where necessary for fluency, brevity or confidentiality, words have been deleted (indicated by ‘…’), whereas vocalisations including ‘um’ and ‘ah’ have been removed. Furthermore, square brackets are used to add explanation where necessary.

## Results

Three overarching themes summarised the common experiences of imprisoned mothers related to giving birth and rearing a young child. The first theme encompasses mothers’ feelings about being pregnant and giving birth while in prison. The second theme reflects mothers’ concerns related to having a young child living with them in prison. The final theme discusses mothers’ feeling of being disempowered to enact their mothering role in the existing correctional system. This theme consists of two subthemes. The first subtheme includes mothers’ perceptions around being restricted in their mothering role in a prison setting. The second subtheme encompasses challenges faced by most mothers in retaining their motherhood identity when their children are living outside prison.

### Theme 1: Imprisonment adds vulnerability and isolation during pregnancy and childbirth

Mothers described a deep sense of loneliness and were worried about themselves and their children during pregnancy and childbirth. Mothers shared experiences where they had felt that their pregnancy related needs were not considered and they were not informed or supported to address their needs during pregnancy and birth, making them feel vulnerable and isolated. These experiences included, for example, being housed in with women having a history of involvement in violent offenses, receiving inadequate or inappropriate nutrition (e.g., for women with gestational diabetes or anaemia), lack of pregnancy multi-vitamins, and difficulty accessing antenatal care because of complex administrative procedures. Mothers said they had limited information and education on perinatal health issues in prison, including access to antenatal appointments and nutrition. One mother said, “*I’m a first-time mum they* [prison] *had no antenatal classes… There wasn’t any offering to go to an outside antenatal class… So pretty much all the knowledge that we could get was from the books that were available in the library.*” [Centre4, GD6].

Several mothers appreciated the weekly- prison-based antenatal check-up and postpartum newborn care provided by a midwife; however, they mentioned that the service was irregular, required lengthy paperwork, and mothers ended up waiting long hours to get the service. Some mothers described being without adequate information about what to expect for birth, breastfeeding, and newborn care, and feeling distressed and out of control. A mother verbalised, *“What their* [mothers] *rights are or how they* [mothers] *go about having their children in* [prison] *or anything really. They* [mothers] *just get told nothing. You’re just left in the dark and there’s no one you can go and ask*.” [Centre2, GD1].

The lack of a support person during labour and birth, or in the days and weeks after birth, was a frequent discussion point. Many mothers believed that having someone on their side was essential to enhance their birthing experiences and they want this to be continued for some time after the birth of their babies. However, not all mothers received approval to have their nominated person at the time of childbirth, because of security reasons, incomplete paperwork, or other prison policies. Therefore, some mothers had to give birth and care for their newborn child on their own without support from their family and/or friends, which they found emotionally overwhelming. One mother described, “*You’re allowed to have one support person in the room with you. They have to leave after the baby’s born. If your child has to stay in hospital for whatever reason, it’s you* [alone] *that stays, if you’re allowed to…”* [Centre4, GD8]. Similarly, another mother stated, *“…you’ve got to deal with it yourself. Whether that’s waking up in the middle of the night to make a bottle or just needing to talk or bub won’t sleep or something like that.”* [Centre3, GD1].

Several mothers reported that the counselling services available in prison are mostly for substance use, self-harm, and suicide; and that there was no counselling for perinatal health needs, including mental health. Some mothers mentioned limited consideration of their physical and emotional needs during tough times, such as experiencing a miscarriage and/or stillbirth. One mother stated, “…*the seats that we get to have to sit on over in secure* [units] *are hard little seats, and I couldn’t sit on it.*“ [Centre1, GD1]. For some mothers, the only support they reported having while in prison was from other women in prison with them.

Imprisonment forced some mothers to endure their pregnancies in isolation, with inadequate maternal health care and support, and uncertainty about whether they would be able to have their children with them in prison or not. For mothers who do not have an approval to have their newborns with them in prison, their babies were removed immediately after birth, and they lost their opportunity to build a bond with their child. “*Particularly young mothers when they get their newborns taken, they’re going to not be able to bond with their children*.” [Centre1, GD1]. Some mothers described the profound emotional suffering of not being able to have photographs at the time of a child’s birth, being separated from their newborn baby, and returning to prison alone. One mother even talked about choosing to terminate a pregnancy to avoid the pain of separation from her baby, “…*what’s better? Having an abortion now, or getting attached to a baby and then having it ripped from you?”* [Centre1, GD3].

The Australian Birth Charter sets out recommendations for supporting mothers in prison to continue breastfeeding or provide breastmilk for their babies, irrespective of whether their child(ren) is with them or in the community (Capper & Baldwin, 2020). However, some mothers in our study described a lack of support and information regarding expressing and providing breastmilk for their babies living in out-of-care arrangements, as this depends on caregivers’ willingness and availability to come to prison and collect expressed breastmilk. For example, one mother explained that despite efforts to give her child ‘the best’, she was not sure whether the baby’s father was collecting her expressed milk from the prison every day and providing it to her child. Another woman mentioned that “…*it wasn’t them* [Child Safety] *that picked up the breastmilk, my mum would come and pick it up and then take it to the office, they weren’t proactive on picking it up.”* [Centre1, GD3]. This mother worried that it would be problematic for her mum if she needs to do this on a long-term basis.

Despite efforts to address the reproductive health needs of mothers, in listening to these mothers’ experiences, it seems there are still inadequacies in addressing those needs in the existing correctional system. Health-based policies are not yet fully fit for the purpose of addressing maternal health needs.

### Theme 2: Concerns around having younger children in the prison environment

Most mothers acknowledged that having their child with them in prison instilled them with hope, a sense of purpose, and direction in their lives: “*I’ve been a mother for so long that without actually having my children, I lose my purpose for life*.” [Centre2, GD4]. For most mothers, developing secure mother-child attachment was the most critical issue and they considered parental support units as providing a good foundation for building a secure attachment and ensuring diligent care of their young children. Gaining approvals to have their children in prison required several complex administrative procedures to be completed and mothers felt that they did not get adequate and relevant information to help them through this process. What was striking was the variations in experiences described by mothers, with some experiencing no difficulties and others reporting extremely lengthy and complicated processes with futile results. One mother recalled her positive experience, “*I think that process was really smooth for me. There have been other mums that it hasn’t been that smooth for. I know other mums that have had to wait weeks and months*.” [Centre4, GD8]. On the other hand, another mother, who described her inability to bring her child to prison despite several efforts, said she was not provided with an explanation for refusal of her request. Some mothers, predominantly those from remote Indigenous communities, perceived that prison policies and procedure discriminate and segregate mothers. They suggested that making the application process explicit and transparent and ensuring support from prison staff in preparing their applications would help to clarify misconceptions.

Despite most mothers having a desire to have their children with them in prison, they were concerned that their children raised in prison would be alienated from the outside world. One mother stated, “…*he’s* [her son] *been in there* [in prison] *for two-and-a-half years. He’s going to be institutionalised*.” [Centre2, GD3]. Most mothers were worried of the potential developmental consequences of early institutionalisation for their children raised in prison, including difficulties in forming new relationships and a variety of cognitive, language, and behaviour problems. Several mothers mentioned of feeling missing out as that they would not have photos or mementos of their children while growing up in prison. One mother expressed that when her child asked about her baby photos, she does not want to say that she does not have any photos “*…because you* [her baby] *were born in prison*.” [Centre3, GD1].

Some mothers attributed the restricted prison environment and limited resources as potential reasons for delayed development milestones of their children. All mothers described a lack of, or limited access to, stimulating activities in the prison setting which would negatively impact the physical and psychosocial development of their younger children. For example, mothers noted limited availability of books, age-appropriate play materials, and opportunities to interact with other children in prison. In one centre where there was no library facility for children, to order books mothers were required to supply a list of children’s books to be ordered without a catalogue or booklist. This would be challenging for mothers with poor literacy and limited exposure to the outside world. It highlights the importance of considering the unique and differing needs of mothers in prison.

Some mothers also voiced that keeping a child in prison can be a significant financial strain, particularly for mothers who do not have supportive family members and friends outside. Mothers mentioned that the prison policy that requires mothers to be able to bear the costs associated with having their young children in prison, such as buying child’s clothes, foods, nappies, and other essentials, creates additional burden as most mothers come from a low socio-economic background and do not receive/earn income or government benefits while imprisoned. As a result of this, some mothers, particularly single mothers with limited financial and social support, chose to keep their children in outside care arrangements. Overall, most mothers believed that having a child in prison can be a positive step in strengthening maternal child bonding but were concerned about the possible detrimental impacts of co-imprisonment on the overall development of children and their integration to society.

### Theme 3: Disempowered to undertake their mothering role in prison

Whether their child was with them in prison or outside, all mothers expressed feeling disempowered to undertake their mothering role. Mothers experienced a sense of powerlessness and being controlled, and their responses are broadly grouped into two subthemes; (i) Restricted mothering within custodial rules and regulations; and (ii) Mothering from inside - struggles in keeping their maternal identities intact.

### Theme 3.1 Restricted mothering within custodial rules and regulations

Mothers discussed several instances where they felt disempowered to undertake their mothering role in the strict prison context, as they lacked autonomy in decision-making and felt scrutinised and judged by correctional officers and other mothers in prison.

Mothers mentioned that they find it very disappointing and painful as they could not exercise their autonomy even for making decisions on matters related to their children’s daily lives, such as choice of food, playing or sleeping times, taking their children to the doctor, or selecting books to read together. One mother explained that “*Before coming into prison we have a lot of involvement and control over what’s happening with the children and stuff like that… You can pick what clothes they need to wear, what medication, things that you want. What formula that you* [want]…*the jail does restrict what things can be bought*.” [Centre 4, GD6]. While every mother and child receive the same treatment and service in the correctional setting, some mothers felt that this might not address diverse needs of children in prison and were concerned that children are forced to fit into prison’s routine. With limited options for baby foods in prison, mothers were worried about nutritional sufficiency and potential allergic reactions that may occur when foods are introduced later into the child’s diet.

Several mothers recounted stories about feeling helpless when their concerns about their child’s health were ignored or not taken seriously by some correctional staff who often disregarded mothers’ requests and made decisions based on their own judgement. Mothers felt that this further disempowered them. In one example, a mother described her repeated, but ignored, attempts to get medical attention for her child, until the child developed a high-grade fever and had to be taken to the hospital emergency department for treatment. Another mother explained, “*when she’s sick, I can’t go and see the nurse because they don’t… They’re only here for the prisoners. So, there’s no actual child nurse*.” [Centre4, GD6]. Not having a child health nurse or primary healthcare services for children in prison sometimes leads to a delay in seeking hospital treatment. This is often due to the associated administrative procedures to seek care outside of prison, which is more concerning at the time of medical emergencies. Because of lengthy paperwork and procedures to get into hospital and anticipation that their requests would be rejected by correctional staff, mothers indicated they sometimes opted not to report their children’s health issues. Mothers felt that the prison setting limited their abilities to show love, care, and support to their children.

Mothers expressed being constantly monitored and judged which made them feel as though they were under continual pressure to prove themselves as a good, caring mother. Failure to provide such an impression or conform to the prescribed behaviours might deprive them from the opportunity of having their child with them in prison. One mother stated, “*If you have your child in here, then they – that’s what they pull over your head. You can’t do – like you’ve got to do this, this, this and this otherwise they take him away.”* [Centre4, GD5]. Mothers’ attitudes highlighted a lack of trust in the correctional system and its staff. This lack of trust was attributed to the perceived absence of concern and empathy shown by correctional staff and fears that their needs would not be considered or that they would be labelled for whatever wrong they had done in the past. The structured prison environment and existing rules and regulations served as an example of an institutionalised form of coercive control, making mothers feel disempowered.

### Theme 3.2 Mothering from inside - struggles in keeping their maternal identities intact

Not all mothers could or wanted to have their children with them in prison. However, whether their child was in informal or state care arrangements, most mothers expressed a desire to retain their active mothering role and resume this once they are out of prison. This subtheme discusses the worries that imprisoned mothers had about their young children living outside of prison. Firstly, most mothers were concerned that imprisonment made them powerless, and they lost their involvement in their children’s upbringing. Secondly, it was difficult for most mothers to maintain regular and meaningful contacts with their young child, despite their belief that maintaining frequent contacts enables a child to adjust to maternal incarceration, reduces emotional trauma of separation for both mothers and their children, and fosters mother-child relationships.

It was particularly upsetting for mothers when they were unable to undertake active roles in their children’s lives and most mothers felt that, if this continued, their identity as a mother would be forgotten by their children and families. Most mothers were not involved in decision making regarding who would be the caregivers of their children outside prison nor were they asked about their preferences. It was evident in the discussions that most mothers preferred informal care arrangements for their children with a trusted friend or relative, preferably grandparents, as they felt this would make the child feel loved and secure and they could contact their children easily. One mother stated, “*He’s* [her son] *never going to go without, he’s always going to be fed, he’s always going to have a roof over his head, he’s always going to be clothed…*” [Centre4, GD3]. However, some mothers were concerned about the parenting styles of their elderly parents. They felt their parents might use disciplinary measures contrary to their own parenting styles or that children might miss out on extracurricular activities, such as early swimming lessons or visits to the park. One mother was worried that her elderly parents might not feel the need to take her son, who has autism, to a speech therapist. Another mother expressed that her own child had become attached to her grandmother and that being distanced from her child made her feel she had lost her purpose in life. She mentioned, *“When I saw her at playgroup, I felt like she didn’t even want to be with me. I remember her so happy and then all of a sudden, she’s just crying, wanting to be with everyone else. It broke me.*” [Centre1, GD2]. There were some mothers who mentioned that when the relationship between a mother and a caregiver is fractured, the mother completely lost contact with their children, and as a result knew little about their children’s preferences, behaviours, and daily routines. With imprisonment, mothers felt they have little or no capacity to engage in or alter decisions in a child’s life, which makes them feel that their maternal identity is being compromised.

Maintaining connections with families and children outside was considered a vital aspect of maternal identities by most mothers. Most mothers felt that telephone contact was not the best choice for infants and younger children as they lack verbal and cognitive abilities required to engage on the telephone, and often rely on facial expressions and gestures to communicate. While mothers preferred face-to-face contact with their children through prison visits, they were worried that younger children might find the entry procedures, including passing through a security gate fitted with alarms and sniffing by dogs, to be scary and stressful. Furthermore, the prison did not have child-friendly spaces and their visits were supervised by prison staff. Mothers expressed that non-contact visits were not suitable as it would be harder for younger children who do not understand the prison’s rules and regulations and the reason for not being allowed to touch their mothers. They felt that the child may end up being confused and emotionally upset. One mother detailed the challenges she worked through to stay connected with her child, *“…my mum has seen the process of when we’ve had the glass visit and the impact that that put on the child. Then my mum had to go home and build them up, make them feel better. So, it was quite even hard for my mum to actually do that too because she was - it made her emotional too.”* [Centre4, GD6].

Some mothers discussed remote videoconferencing as an alternative to face-to-face visits, as it allowed meaningful interactions, including interacting with children in their home setting, assisting children with their homework, or reading a book for them, while also being a low-cost option. A mother shared how remote videoconferencing would assist her in continuing her mothering role in prison, “*If I had access to weekly video calls with my child where I could read books to them, and we could talk and we could play and things like that, my children never fail to have 10 billion things to show and tell with me on our video call once a fortnight.”* [Centre4, GD8]. However, mothers reported some barriers in using videoconferencing, including the willingness and readiness among the caregivers to facilitate the process and lengthy administrative procedures. Caregivers need to have knowledge of the technology, access to devices, and a secure internet connection: “…*your children are with the grandma' of the child, your mother, she may be older, and she might not know how to work technology*.” [Centre4, GD1]. Similarly, one mother stated that, “*I’ve put in the form for the video conference but the timeframe to get that approved takes up to six months. They have a lot of connection issues with video links as well.”* [Centre4, GD6]. Most mothers recommended a need of more resources in the correctional centres and support to caregivers in processing the request and using the technology and devices.

As most mothers intended to reunite with their children upon release, it is of paramount importance to strengthen and improve family interactions during incarceration. Analysing mothers’ responses indicated that allowing younger children to remain with their mother in prison was a step in the right direction; however, there were some systemic barriers that restricted mothers in enacting their motherhood role in an engaging and empowering way. Some systemic issues frequently raised in the discussions centred on the correctional system not being designed appropriately for mothers and younger children, limited consideration of the varied needs and experiences of imprisoned mothers, limited human resources, complex bureaucratic procedures, and the institutional culture of corrections.

## Discussion

The present study examined how pregnancy and mothering a young child was experienced by currently imprisoned mothers. Mothers provided accounts of challenges associated with being pregnant and undertaking a mothering role in the prison environment, including, (i) reproductive health needs not being prioritised in the current prison system; (ii) mothers being uncertain about what is in the best interests of their children; and (iii) mothers feeling disempowered to enact their motherhood role. The discussion focusses on two main issues that evolved from the workshops with mothers: struggles that imprisoned mothers face in navigating motherhood; and, aspects of correctional centres or procedures that could be changed to improve mothers’ experiences in connecting with, and mothering, their children.

### Struggles in navigating motherhood while in prison

The United Nations (UN) Bangkok Rules recommend that all women, regardless of their incarceration status, deserve to have a healthy, safe, and dignified pregnancy and birth (Capper & Baldwin, 2020; United Nations 2011). Mothers should receive care that is grounded in the “woman-centered approach”, which means women’s needs should be central, and their plan of care should be individualised considering their specific circumstances and their right to choice and dignity (Thomson et al., 2022; Baldwin et al., 2020). However, the experiences of mothers in this study exemplify, and are consistent with, other studies showing that correctional policies and procedures were mostly designed at a time when female incarceration was low, and that prisons are still not equipped to address the reproductive healthcare needs of imprisoned mothers and their children (Bronson & Sufrin, 2019; Kelsey et al., 2017; Shlafer et al., 2019). Similar to the observations made by mothers in the current study, other US-based studies concluded lack of appropriate nutritional and accommodation arrangements to address the changing needs related to diet, sleep, rest, and physical activity of pregnant women (Ferszt & Clarke, 2012; Kelsey et al., 2017). The Bangkok Rules recommend that pregnant women in prison should receive quality antenatal care equivalent to that available in community (UN, 2011). However, findings of this study are consistent with other studies regarding inadequate antenatal care, difficulties in accessing outside antenatal services, and lack of formal support to optimise pregnancy outcomes (Ramirez et al., 2020; Baldwin et al., 2020). Mothers in this study wanted emotional support, counselling, and access to family-based programs during their perinatal period. However, few programs were available and eligibility criteria were strict, thus, only a small fraction of women receive services and many mothers, particularly those on remand or short sentences, remain deprived of relevant maternal services.

Resonating with findings from a study done among women imprisoned in the US state prisons, mothers in this study felt that the correctional system often adds layers of punishment to mothers (Kennedy et al., 2020). Mothers in custody were often previously the sole caregiver of their children and spent much of their time thinking and worrying about the whereabouts of their younger children who were outside (Shlafer et al., 2019). Mothers who do not get approvals or do not want to have their children live with them in prison, usually preferred their relatives (mostly their mothers or sisters) to take caregiving responsibilities for their young children (Glaze & Maruschak, 2010), anticipating that this would enable them to maintain connections with their children and regain custody after their release. Our study expands on this research and adds critical insights on the challenges of maintaining motherhood identity when children are in outside care arrangements. In most instances, mothers were unsure of how their children were being taken care of outside and were worried about regaining custody of their children after their release from prison, as most of them did not have secure housing and income support. Currently, few resources are available to help mothers understand the legal implications of such informal placements of their children and the demand of housing far exceeds the supply, leaving some women with no choice but to return to a violent partner or become homeless.

This study adds to existing debates on the co-placement of younger children with their mothers in prison. Though, most mothers in this study felt that residing together with their young child would address the negative impacts of separation and motivate them to change for a better future (Kennedy et al., 2020; Pendleton et al., 2020), they felt that prisons do not offer a healthy environment to help children grow to their full potential, which is consistent with other studies (Nuytiens & Jehaes, 2022; Walker et al., 2021). Similar to previous studies, this study concluded that mothers in prison felt disempowered and were unsure of how to consider their child’s “best interests” while making decisions about their care (Abbott et al., 2020; Baldwin, 2018). Mothers in this study felt that existing correctional structures and policies, based on principles of punishment and correcting deviant behaviour, lack of control and agency to mothers, and limited information sharing reduced their autonomy and authority as mothers.

Our finding that all mothers wanted to have regular and meaningful contact with their children to help them keep their maternal identities intact has been echoed in other studies as well (Aiello & McCorkel, 2018; Poehlmann et al., 2010). Mothers preferred in-person visits where they can hold and directly interact with their infants and young children in child-friendly visitation environments (Horgan & Poehlmann-Tynan, 2020; Haverkate & Wright, 2020). However, consisted with prior literature, several systemic issues that inhibit in-person visits are further elucidated in this study, mostly by Indigenous mothers and mothers from rural and remote communities and included the distant location of the female correctional facilities, high cost of transportation to get there, strict security procedures, and lack of child-friendly spaces in prison (Poehlmann et al., 2010; Schubert et al., 2016). In accordance with other studies, this study concluded that telephone or written letters were the most used modes of communication in custodial settings but were unsuitable for younger children as they lack cognitive and verbal abilities to effectively use them (Minson, 2019; Horgan & Poehlmann-Tynan, 2020).

### Addressing challenges commonly experienced by imprisoned mothers

The correctional system acknowledges that mothering is important, but it is critical to go a step further to not only acknowledge but improve mothers’ experiences during pregnancy and the early years of mothering when they have additional needs. From the direct experiences of mothers in the current study and supported by literature, the following recommendations are made to address the conflicts in the existing system and support imprisoned mothers in undertaking their mothering role actively.

### Alternatives to incarceration: doing things differently

There seems to be an increasing and widespread recognition of gender-specific and non-custodial responses for pregnant women. Australian jurisdictions have shown support for justice reinvestment that involves reallocating funds from building prison infrastructure to creating community-based diversion programs (ADCQ, 2019; Bartels et al., 2020). Exemplars of good diversionary practices in Australia are The Home Detention Integrated Services Program in South Australia, Court-ordered parole in Queensland, and Victoria’s Assessment and Referral Court (Productivity Commission, 2021). Although these programs have shown some cost-savings and reduction in reoffending, they have strict eligibility criteria, ruling out those charged with serious or violent offences or sentenced to less than two years in custody (Productivity Commission, 2021). As a result, only a small fraction of the prison population can utilise these services but there are no formal records of number of pregnant and parenting women who have accessed them, which could be an important avenue for further research. The increasing number of women in prison in Australia suggests insufficient implementation of non-custodial options and increased incarceration of women offenders (UN, 2011). This study supports the need for expansion of eligibility criteria and significant shifts in sentencing practices to allow more mothers to reside with, and parent, their babies/young children in community-based residential arrangements, while accessing support for housing, parenting, and abstaining from substance use (ADCQ, 2019; Bartels et al., 2020). Being able to reside in a community facility with their child/ren would eliminate some of the concerns expressed by mothers in our study for their children, including, institutionalisation, disempowerment, limited development opportunities, and poor social relationships.

### Targeted approach to support mothers in enacting their mothering role

Not all mothers in prison are the same; indeed, not all will have similar experience with pregnancy, birth, or rearing a young child. There is a need to make targeted and concerted efforts to provide incarcerated mothers with comprehensive reproductive healthcare and support during birth and the post-partum period in prison (Shlafer et al., 2019). Evidence shows that enhanced perinatal care provision had improved perinatal outcomes, including adequate prenatal care and decrease in a preterm or caesarean delivery (Bard et al., 2016). Examples of these are the doula support program that provided pregnancy, birth, and parenting services to mothers incarcerated in several prisons across the US (Shlafer et al., 2019), and a UK-based charity, called Birth Companions, that provides antenatal and parental courses in various prisons in England (Thomson et al., 2022). These programs have highlighted the need to develop meaningful connections with mothers in prison, deliver individualised care, and facilitate access to other support services (Shlafer et al., 2019; Thomson et al., 2022). Although promising, given the uniqueness of individual and family circumstances, there is no-one-size-fits all approach. Continuous efforts should be directed towards development and implementation of evidence-based models that are tailored to the needs of imprisoned pregnant women. Several systemic barriers, including staff shortages, limitations in prison’s resources, access to reproductive healthcare, and rigid security restrictions, as reported by most mothers, need to be addressed.

Although most prisons in Australia have dedicated facilities for approved mothers to have their child with them in prison, which has the potential to enhance mothers’ bonding and parenting knowledge and skills (Shlonsky et al., 2016; Tremblay & Sutherland, 2017), there is a need to adapt correctional policies to enable mothers to actively undertake their roles as mothers. There is evidence that involving mothers in care planning and decision-making for their children improves their satisfaction (Trotter et al., 2017), but mothers in our study revealed that they were not usually involved in decision-making processes. At times, and consistent with a previous study (Bronson & Sufrin, 2019), mothers in this study spoke of their frustrations with the correctional system and its staff who they believed were not supportive, fair, and empathetic to their needs or those of their young children and did not provide them with relevant information to make informed choices. This can be due to the struggle that some prison staff face while keeping a balance between maintaining custodial rules and responsibilities and providing individualised care (Pendleton et al., 2020). Training prison staff about needs of mothers and young children and effective communication skills would help them in maintaining trauma informed conversations with mothers in prison and responding effectively to their needs. Instead of making assumptions about the needs of mothers or adapting evidence generated from studies conducted among men and/or general women, it is important to continually explore mothers’ choices and provide them with cultural- and context-relevant information to facilitate decision-making. Mothers’ experiences while rearing a young child in prison should be considered at all levels, including architectural design of prisons, staff training, and prison policies and regulations. Mothers need to be supported to meet the developmental needs of their children, including positive parenting, healthy nutrition, early learning opportunities, and safe living, whether their young children live with them in prison or are in other care arrangements.

### Adopting good practices to improve maternal-child communication

Some correctional policies and practices that severely impact mothers’ capacity to maintain contact with their younger children need to be reviewed and improved. These include non-contact visits, phone calls during weekdays, limited time allocated for phone calls, and limited options for family visits. Correctional centres should ensure regular and developmentally appropriate contact between mothers and their children is available and accessible. There are several good practices that are being implemented globally, which have the potential to support maternal-child relationship building, without compromising overall prison security. Examples include provision of free transportation services to facilitate prison visits (e.g., Get on the Bus program of California; Children’s Supported Transport Service by Shine for Kids, Australia); audio recording of stories for children (e.g., The Women’s Storybook project of Texas; ‘Story Time’ program by Shine for Kids); regular extended visitation in child friendly areas (Bard et al., 2016; Paynter et al., 2020), and video conferencing (Flynn et al., 2021). The present study supports the use of remote videoconferencing to offer flexibility and increase access by addressing challenges associated with visiting, notably distance and cost (Cramer et al., 2017; Horgan & Poehlmann-Tynan, 2020). Furthermore, mothers felt that virtual contact prevented their young children from being exposed to the prison environment, and allowed them to see, talk, and interact meaningfully with their children in a familiar home setting (Flynn et al., 2021; Horgan & Poehlmann-Tynan, 2020).

While video calls and story recordings can be great resources for maintaining family connections, several challenges have been reported in literature, similar to our findings. These include limited access to electronic devices and the internet, lack of competency to use them effectively among caregivers and mothers, and lengthy administrative/permission procedures (Flynn et al., 2021; Horgan & Poehlmann-Tynan, 2020). Adopting strategies to help mothers in building their skills in using virtual modes of contact, such as use of video tutorials or visit coaching, ensuring support to co-ordinate and set up videoconferencing for families in communities, and training prison staff on ways to effectively use technology could help in improving mother-child engagement (Cramer et al., 2017; Flynn et al., 2021). Furthermore, working alongside mothers and caregivers to decide on the best approach to contact their children, considering the needs of children and family, is recommended.

### Limitations of this study

We note some methodological limitations of this study. First, mothers were asked in general about their needs while in prison and after release; there were no specific questions on mothers’ needs during pregnancy and birth. However, in a situation where mothers raised these issues, we probed them further and created a space to talk about those issues. Therefore, we acknowledge that the depth of information regarding pregnancy and birth in prison might be limited; however, given the relative lack of empirical research in this context, this study nevertheless makes an important contribution to better understanding of experiences of mothering young children while in prison. Second, as this study explored subjective experiences of mothers in prison, there might be a possibility of self-reporting bias. As a response to the coronavirus pandemic, correctional settings had suspended in-person visits and introduced virtual visits. This could be one of the reasons for mothers commenting on negative experiences related to pregnancy and motherhood in prison. However, other studies discussed previously also support negative experiences associated with mothering in prison. With ongoing changes in prison policies and regulations, there is a continual need of studies exploring mothers’ current experiences. For participant confidentiality reasons, we were unable to seek any verification of mother’s accounts of events with the correctional agency. Third, the sample may not be representative of the entire population of mothers in prison, as there is a possibility of self-selection bias. Mothers who felt disconnected from their mothering roles or felt detached from their children due to their imprisonment may have been less likely to volunteer to participate in the study. However, the sample was heterogeneous, allowing a good mix of mothers from both high and low security prisons, from different age groups, as well as from Indigenous and non-Indigenous backgrounds. It is well documented that Indigenous women and culturally and linguistically diverse women have specific programming and service needs (Bartels et al., 2020). However, as data were collected in a group setting, it was not possible to explore how experiences of being pregnant and mothering a young child differ between Indigenous and non-Indigenous mothers. Another limitation of the study is the inclusion of only those mothers who were able to speak in English. Further research is warranted to explore the needs of Indigenous and culturally and linguistically diverse mothers and the way their cultural identity and connectedness can impact their mothering experiences while in prison. Lastly, it is acknowledged that workshops may not have allowed sufficient time to delve more deeply into the experiences of mothers in prison. However, rich data were still obtained as mothers were enthusiastic in sharing their views and actively participated throughout the discussions.

## Conclusion

Mothers’ accounts of their needs, highlighted in this study, will contribute to the co-creation of a service delivery model for incarcerated mothers and their children in Queensland. Being a mother has existential meaning for women; however, the experience of motherhood is complex and profound for mothers in custody. Pregnancy and mothering a young child in prison adds several practical, emotional, and physical challenges to imprisoned mothers and the correctional system and policies, which were predominantly designed for men, are not yet fit to address their varied and complex needs. The present study provided new insights, in that undertaking a mothering role in prison created conflicting situations for most mothers where they felt unsupported and confused in deciding what would be best for their child/ren. Mothers’ experiences highlighted the need to revise or replace some correctional policies and procedures, so that mothers’ needs, and those of their children, can be better addressed and they are supported to continue an active mothering role in their child’s life. Increasing access to information and support services would help mothers in deciding what is best for their child considering their circumstances. Adopting non-custodial measures for mothers with young children, supporting mothers in using age-appropriate methods of contact, and ensuring that mothers get opportunities and support in prison to exercise their motherhood independently, can facilitate the process of personal transformation and improve outcomes for both mothers and their children.

## Electronic supplementary material

Below is the link to the electronic supplementary material.

Supplementary Material 1

## Data Availability

The datasets generated and/or analysed during the current study are not publicly available due to the confidential nature of the study but are available from the corresponding author on reasonable request.

## Footnotes

1 In Australia, the term ‘correctional centre’ is used to formally refer to the correctional facility in which a person is held. This term is used interchangeably with the more common term, ‘prison’. Both these terms will be used throughout the paper. Unlike some other countries, such as the United States, Australia does not have separate jails and prisons. Rather, the term ‘jail’ is used as a slang word for ‘prison’ or ‘correctional centre’. In Australia, all individuals sentenced to custody or held on remand will be housed in a correctional centre/prison.

## Declarations

Committee (GU Ref No.: 2020/777). All participants provided informed written consent to participate in the study.
