# Supplementary material for: Navigating pregnancy and early motherhood in prison: a thematic analysis of mothers’ experiences
Source: Health Justice. 2022 Oct 29;10:32. doi: 10.1186/s40352-022-00196-4 (PMC9617046; doi:10.1186/s40352-022-00196-4)
Supplement: Supplementary file 1 — Supplementary Material 1 [file 40352_2022_196_MOESM1_ESM.docx]

**Feedback from reviewers**

|  | **Reviewer’s comments** | **Authors’ responses** |
| --- | --- | --- |
|  | **Reviewer 1**: This paper covers the important topic of pregnancy and motherhood experienced by 75 women in Australian prisons. The authors thoughtfully point out that mothers’ who are in prison have complex needs that are often overlooked and the challenges mothers face while engaging with their children during incarceration (inside or outside the institution). There are only a few general concerns with this piece that require attention: expanding on the literature presented in the Background section and connecting it to the Discussion section better; defining terms/phrases and providing more specific examples when concepts/situations are discussed throughout the paper; be more explicit about the policies/practices of the correctional centers that interfere with women’s abilities to mother while incarcerated; and contextualize the quotes in the Results section more thoroughly to better grasp the situation women are discussing. Some helpful literature is listed below as well. Otherwise, the takeaways of this paper fit the scope of the journal; the field of corrections along with feminist scholars will surely benefit from its contribution. I sincerely enjoyed reading this paper | |
| **A.** | **Background** |  |
|  | The authors clearly know the literature base, but this section could be clarified in a few places and made more specific at points. A lot of the literature is in the Discussion section already (Lines 683-688 are great)! To start, it’s an overgeneralization to say that children are allowed to reside with their mothers in prison globally. It might be helpful to give some examples of where this is indeed the case.  It’d also be important to note that the research on mothering in prison is actually quite robust (e.g., Casey-Acevedo et al., 2004; Hobler, 2001; Houck & Loper, 2002; Hughes & Harrison-Thompson, 2002; Laughlin et al., 2008; Poehlmann et al., 2008; Snyder et al., 2002; Wells, 2019), but focusing on women’s perceptions/preferences is where it is lacking. Make this point stronger!  Additionally, children are obviously discussed a lot in the Background section, but the age ranges for this group, particularly “dependent” or “young” children (Line 35 & 46) is not defined until later on in the paper. It would be helpful to set this up from the start.  Also, it is stated that “early motherhood” is not well-researched, but does this mean first-time mothers or those who have been mothers before but have young children? Or first-time in-prison mothers?  Further, describing health and wellbeing of mothers is important but what does the research say is encompassed in these concepts? How are they operationalized? This could mean many things, especially in terms of pregnancy/birth/postpartum. If not well researched, state that and indicate examples (e.g., X) of items to consider under these concepts.  Lastly, the final paragraph of the section discusses contact between mothers and children being positive, which is mostly true, but there is some literature that would indicate mixed findings. For example, mothers’ sometimes experience negative impacts of communication with children such as increased misbehaviour while incarcerated after writing letters to children (Benning & Lahm, 2016). | Thank you for your comments.  Some countries where mothers are allowed to have their young children with them in prison are mentioned in the Line no. 132-33.  Preferencing the voices of mothers and their experiences of pregnancy and mothering in prison (Line no. 37-40) was the focus of this paper. We have included some additional sentences to make it clearer where existing literature is lacking (Line no. 41-55; 178- 181; 210- 220).  In most literature, dependent children refer to children under 18 years. The needs of mothers of young children (less than 5 years) are likely different to those of mothers of school-going children or teenagers. This study aimed to explore experiences of mothers who have children aged 0 to 5 years as this is the most vulnerable period in a human’s life and growth and development at this stage predict overall development over the life course (Line no. 104-106; 190-191). Furthermore, in most prisons in Australia, children are allowed to reside with their mothers in prison until they start their school, if mothers meet the eligibility criteria (Line no. 205-207). The age range of children considered in this paper is now mentioned at the start (Line no. 4, 43)  Thank you for your comments. We have revised the sentences to make it clearer. (Line no. 46)  In this paper, we haven’t focused on a particular health condition, rather mothers were asked about their overall health and wellbeing during pregnancy and post-birth.  Thank you for raising this point. We have now included this in a last paragraph of the ‘Background’ section (Line no. 145 –149) |
|  | *A bigger question to consider: Are you only focusing on/researching with women that identify as women and mothers or are there folks with other gender identities in the sample that are also mothers? If so, that may change some language throughout. If the former is true or it is unknown, then stating that in the methods might help to set the parameters of the sample | Thank you. This study included women who have self-identified themselves as mothers (Line no. 211). |
|  | To strengthen this section:  • Add more examples or (e.g., X) to flesh out what is meant by certain concepts/points.  o Ex: Line 39-41; health/well-being of mothers and children impacted by maternal incarceration are important, but examples are useful.  o Ex: Line 85-86; instability in a child’s life means what exactly? Provide examples. | This first paragraph has been revised and no longer refers to the health and wellbeing of mothers and children. We later describe these concepts (Line no. 50-57; Line no. 96-103)  We have expanded on the sentence to clarify what is meant by instability in a child’s life (Line no. 80). |
|  | Provide descriptions or explanations of certain terminology in parentheses (e.g., perinatal, antenatal care, prenatal, gestational age, postnatal) as some readers may not know much about pregnancy/birth. Footnote, at the least | Given that these terms are not central to the paper (i.e., they were not operationalised or featured in comparisons), we consider that explaining these terms unduly increases the number of words. |
|  | Add more literature/context on the importance of visitation/communication trends between mothers and children and the health and wellbeing of mothers and children since they are discussed in the results/discussion. | Thank you. We have now expanded our literature review to explain the impact (both positive and negative) of mother-child contacts on both mothers and their young children (Line no. 134 –149). |
|  | Clarify the age ranges for children of study | Findings related to children aged 0 to 5 years were included in the study. It was specified in the background section of the article (Line no. 43, 108, 191) |
|  | Clarify the gap in literature that this study fills | This study aimed to explore experiences related to pregnancy, birth, and the rearing of young children of mothers who are in prison, regardless of whether their children are with them in prison or outside. This study included voices of these mothers which is mostly lacking in the existing literature (Line no. 37-44). Paragraphs under the headings “The Current Study” describe the gap in literature that this study fills (Line no. 151-189). |
| **B.** | **Current study and methods** |  |
|  | The set-up of the study is great, very well-written and easy to read. In fact, line 175-177 is spot on, and it would be helpful to state it earlier on in the Background section.  In the methods and beyond, the authors continue to state vaguely issues with the “policies/practices/procedures of the correctional centres” but do not actually give any examples or specific guidelines that the centers housing the women of study experience. Some examples in the methods section around lines 208-209 would be useful.  Also, for readers that do not know what qualitative data analysis entails, a brief description of the purpose of reflexive thematic analysis would be helpful, even if footnoted. It might also be helpful to provide citations for the methodological choices used such as line-by-line coding (Charmaz, 2006). The last paragraph of the methods section is quite detailed, so that’s great! | Thank you for your feedback. This has been mentioned in the background section (Line no. 37-40).  It was not possible to specify policy and guidelines as mothers were not asked specifically about any single policy or program. This study did not intend to examine particular policies, rather mothers were asked in general about available policies/ programs/ services in prison and what they need to improve their experiences.  Thank you for your comments. We have revised the section as suggested and added some descriptions about reflexive thematic analysis (Line no. 249-265). |
|  | To strengthen this section:  • Line 165-166 – what are mothers/young children’s unique needs? Provide examples (e.g., women have high rates of SMIs). | This paragraph is more about summarising the introduction section. In the introduction, we have detailed the different needs of pregnant women and mothers in prison who have young children, such as antenatal care during pregnancy, coping with separation from young children, increased risk of mental illness, and increased risk of pregnancy complications (Line no 50-57; 96-103; 136-138). To make it clearer, we have added some information on the unique needs of mothers of young children (Line no. 166-168). |
|  | Line 167-168 – maternal identities makes sense, but some readers might ask, “meaning women’s feelings about being a mother or holding that role and identifying with it?” | We have now revised the sentence to explain our use of the term “maternal identity” (Line no. 168 - 170) |
|  | Line 181-182 – what might those outcomes be? Provide examples (e.g., relationship quality? Feelings of connectedness?). | We have now clarified this in the sentence (Line no. 186-187). |
|  | Line 191 – define young. | Revised as suggested (Line no. 43) |
|  | Line 228 – how many outliers? | There was only one outlier. The length of sentence was 108 months (Line no. 224). |
|  | Line 230-231 – are Northern Queensland and South-East Queensland pseudonyms for the correctional center names or just locations of where the centers are generally located? | Northern Queensland and South-East Queensland were used based on the locations of the centres where data collection was undertaken (Line no. 226-227). |
|  | Line 253-254 – were the group discussions transcribed into documents? Was a data management software used? | All workshops’ recordings were transcribed using a professional transcription service. Microsoft Excel was used for data management and coding, while Microsoft Word was used to thematically organise quotes (Line no. 246-248). |
|  | Line 260 – what did the memoing process look like? | There were no rules around memoing and each memo contains researchers’ reflections about the data, the research process, and observations during the workshops. Some sentences were added to clarify the analysis process, including the memoing process (Line no. 259-264). |
|  | Line 264 – trustworthiness and rigor do not accurately capture what is being stated in the paragraph, it sounds more like reliability and validity concerns. Consider changing the subheading. | We have revised as suggested (Line no. 267). |
|  | Line 266 – “Ensured themes were close to the data” is vague, perhaps “direct reflections?” | We have revised as suggested (Line no. 269). |
| **C.** | **Results:** |  |
|  | The results are well-organized! However, some of the supporting quotes are not stand-alone quotes and need a bit more lead-up to help contextualize them or a reiteration after the quote to fully grasp the situation that women are discussing (e.g., quote on Lines 395-396).  When discussing whether women can have their children with them in prison or not, it might be useful to explain the regulations that dictate this decision. What’s the process? If it’s a super complicated process, a flow chart diagram might be helpful. Again, similar to the methods, issues with policies/practices are mentioned, but not what those are exactly. They need to be listed out. For instance, on Lines 432-435, mothers are unable to make decisions about their children’s daily lives regarding food, sleep, play, doctors, etc., but what are the rules in correctional centers that override the decisions of mothers or is it simply up to officer’s discretion? I think this needs to be said explicitly.  Lastly, while recommendations are presented in the discussion, did women themselves offer any recommendations they would like to see implemented? | Some descriptions are added in the revised manuscript to clarify the quotes (Line no. 324-326, 397-401).  ***General process of having a child with their mother in prison:*** Every mother is assessed against the eligibility criteria to determine if she can have her young child with her in prison. Such assessments are done in the correctional setting and decisions are based on findings from the assessment of mothers’ history, her willingness to have her child with her in prison, her ability to take care of the child, the availability of a suitable emergency caregiver in the community, and space and operational considerations at the relevant centre. General managers of the prison make decision about whether to allow baby to live with their mother in prison after evaluating all these findings. Procedures and policies guide this process and decisions are made on a case-by-case basis. It is beyond the scope of this paper to present the policies and regulations that dictate this process; however, we have provided relevant references for those who want to know more on this topic. In this paper, we want to highlight that there was a variation in experiences among mothers who want to bring their babies in prison and some mothers indicated that they were unaware of conditions that preclude them from having their babies with them in prison. However, we did not cross-check case decisions with correctional centres due to maintaining mother’s confidentiality and because their self-reported experiences were the focus of the paper.  We were unable to discuss how decisions about children are made in the correctional setting. This statement (Line no. 433-436) was kept to highlight the situation where mothers felt that they have lost their autonomy and power after coming into prison. We have made some changes to clarify this in the revised version (Line no. 433-436)  Mothers included in the study also provided recommendations for improvements, but as the focus of this paper is on mother’s experiences and needs, it is beyond the scope of the paper to include a section on these. However, their recommendations were considered while designing the new model of support for mothers and their children and will be detailed in another paper. Nevertheless, the results section includes key improvements that mothers reported that they want to be implemented in a prison setting (Line no. 328-329; 378-379; 390-391; 533-534) |
|  | To strengthen this section: |  |
|  | Line 304 – specify by saying most/some women; right now, it sounds like all mothers. | Revised as suggested (Line no. 303). |
|  | Line 305-308 – any quotes to support the statement about pregnancy related needs not being met and changing needs throughout pregnancy/birth? | Quotes related to the inadequacy of antenatal classes (Line no. 315-318), and the lack of support person during and after birth are included in the manuscript (Line no. 334-338); limited consideration of their needs during tough times (Line no. 343-344). |
|  | Line 308 – for “violent offenders” consider changing to “being housed with women who have violent records” for person-first language. | Revised as suggested (Line no. 310 – 311). |
|  | Line 315 – did women describe what information was in the books they were able to access in the library? | No, mothers did not specify what information they got from the books that they were able to access from the library and they were not probed further to specify such information. From their statement it can be deduced that they were talking about antenatal check-up schedule, nutrition during pregnancy, physiological changes during pregnancy and so on. |
|  | • Line 317-318 – was the midwife program a volunteer program? Knowing this might help contextualize the inconsistency of the program being held. | This was a regular prison-based antenatal health program for pregnant women in prison. This has been specified in the manuscript (Line no. 319-320). |
|  | Line 321-322 – are the “their” and “they” in the quote referring to mothers? Quote is kind of confusing | We have added some descriptors in the quotes to make it clearer (Line no. 324- 326). |
|  | Lines 527-528 – regarding contact in correctional centers, was there any mention of issues with staff and video technology, like repeated interruptions or cancelling visitations? If possible, a quote here would be helpful. | We have added some quotes to explain some connection issues with video links at prison (Line no. 529-533). |
| **D.** | **Discussion/Conclusion** |  |
|  | The discussion is easy to read and the conclusion is succinct! I see the UN is cited, but do they have specific pregnancy/mothering guidelines? The UN has guidelines for the treatment of women prisoners, but they might also have some on pregnancy/mothering or the guidelines specific to women may include a section on pregnancy/mothering. Those could be applied in the Discussion section, if they exist.  As stated in the Background section, it would be helpful to add literature on visitations/communication between mothers and children as it could contextualize the finding on mothers preferring in-person visits (Lines 600-602). It is explained that the number of mothers accessing services is hard to know because there are no records of this information, and this an important avenue for future research which should be stated.  Also, it’s hard to follow the argument in Lines 630-631 about sentencing policies being designed for men who have violent criminal records. This needs to be expanded.  Furthermore, it is suggested that assumptions about mother’s needs are sometimes adapted from evidence on “different population groups,” but what does that mean? Which groups and how?  Also, Indigenous women are briefly discussed throughout the piece, but there is not a specific section in the results for their perhaps unique perspective if it’s even captured in the data (maybe for another paper?), nor is there a call for future research on this particular population that is also culturally sensitive to their mothering needs.  Just reiterating, but the correctional system policies that are designed for men that negatively impact mothers and children by ignoring their needs should be listed out to strengthen the argument in the conclusion (Lines 735-737). Lastly, Lines 670-672 are fabulous! | The United Nations has recommended some rules related to the treatment of women in prison and have provided some guidelines for imprisoned pregnant women. This has been mentioned in the Discussion section (Line no. 555 – 557; 568-572)  In the background section, we have included literature on visitations/ communication between mothers and children (Lines 134-149; 179-182).  As suggested, we have included the recommendations on conducting future research to explore number of mothers accessing different services (Line no. 635-636).  We have revised the sentence to make it clearer (Line no. 636-638).  We have specified that the different population groups mean men or general women (Line no. 679).  Mothers’ experiences were recorded in a group setting and we didn’t have responses allocated to each individual. In every workshop, there was a mix of Indigenous and non-Indigenous women, therefore it was not possible to distinguish between experiences of Indigenous and non-Indigenous mothers. Thanks for raising this important point and it would be interesting to explore how Indigenous mothers experience pregnancy and mothering in prison and how their experiences are different to that of non-Indigenous mothers. This has been mentioned under the limitations of the study (Line no. 739-741).  In the conclusion, there is a line that indicates how existing prison policies that are predominantly designed for men are unable to address multiple and diverse needs of pregnant women and mothers of young children (Line no 753-756) |
|  | Lines 551-552 – define what “woman-centered care” is according to Baldwin and colleagues or the adapted interpretation of it. This is a really important topic! | Thank you for your suggestion. We have defined woman-centred care in the discussion section (Line no. 557-560) |
|  | • Lines 554-555 – “when female incarceration was rare” meaning between the 70s-early 90s? | We have revised the sentence to make it clearer (Line no. 562). |
|  | Lines 591-594 – sentence is a bit confusing; consider specifying the items in the list and breaking into multiple sentences | The sentence was revised to make it clearer (598-600). |
|  | Line 617 – “Doing thing differently” needs an ‘s’ on “thing” | Revised as suggested (Line no. 624) |
|  | Line 641-642 – might be useful to discuss postpartum depression here | We wanted to highlight that services should be comprehensive and individualised to improve health of mother during pregnancy, at birth, and after birth. Rather than focusing on a specific issue, such as postnatal depression, we have discussed a whole range of health conditions that mothers are at risk of during these times. |
|  | Line 643-644 – what are pregnancy outcomes? Provide examples | Revised as suggested (Line no. 651-652). |
|  | Lines 691-693 – specify that remote video conferencing is what is being referred to since some video conferencing takes place within correctional centers. | Revised as suggested (line no. 519, 522, 699). |
|  | Sentence starting on Lines 727-730 could be removed. | We have removed these sentences. |
|  | Line 748 – consider changing “outcomes” to “relationships”. | We have retained “outcomes” as it refers to a whole range of measures, including but not limited to relationships, physical health, mental health, social support etc. |
| **E.** | **Other points** |  |
|  | There are a couple minor issues in the paper. For the authors’ consideration:  • Line 13-14 reads awkwardly. Consider “agreed upon…” | Revised as suggested |
|  | Numbers written as “5-10%” should be 5 to 10%, or “3-5” as three to five, 60 – 90 minutes as 60 to 90 minutes, etc | Revised as suggested |
|  | Closed parenthesis is needed on line 57. | Revised as suggested |
|  | Line 201 needs a comma in 3,292. | Revised as suggested |
|  | Line 258 – “agreed codes” maybe missing “upon”? | Revised as suggested |
|  | Line 266-267 – “themes allowed credibility-checks” maybe missing “for”? | Revised as suggested |
|  | Line 378 – “diligent care to their young children” maybe “of” instead of “to”? | Revised as suggested |
|  | Line 541 – “a mothering role in prison environment” maybe missing “the” or “a”? | Revised as suggested |
|  | Line 544 – “enact their motherhood” maybe missing “role” at the end? | Revised as suggested |
|  | Line 600 – consider adding (e.g., citations) since studies are referred to and not directly cited. | Revised as suggested |
|  | Line 607 – consider changing “were” to “are”. | Revised as suggested |
|  | Line 696 – consider adding “them” after “to use”. | Revised as suggested |
| F. | Helpful citations |  |
|  | Around Lines 139-141 – in-person visitation between parents and children during incarceration increases relationship quality between them (Haverkate & Wright, 2020).  Around Line 150 – there are fewer women’s institutions than men’s institutions, so mothers are housed farther away from their children (Bloom, 1996; Genty, 1998; Hairston et al., 2004; Lindquist et al., 2015). This was stated in the Discussion section, but not in the Background section. Would be great in both! | Thank you for pointing out these important studies. We have reviewed and included suggested references wherever appropriate, such as Benning & Lahm, 2016; Haverkate & Wright, 2020; Casey-Acevedo et al., 2004 (Line no. 138, 181,608).  Revised as suggested (Line no. 142 – 143) |
|  | Overall, this study fills a necessary gap in the literature base by illuminating the perspectives of actual mothers as they navigate pregnancy/childbirth, parenting roles, and connections with children while incarcerated. The revisions suggested are minor and the comments are simply for the authors’ consideration. Well done! | |
|  | **Reviewer 2:** Thank you for the opportunity to review this manuscript. It is generally a clear and well-written paper on an essential topic of mothers' experience of pregnancy and early motherhood during incarceration. Specifically, the purpose of the present study was to explore imprisoned mothers' needs while in custody and post-release and the kind of support and system changes required to meet those needs. I have read the manuscript with great interest. Most studies on these subjects were focused on the perspective of children or mothering school-age children, while this study focused on pregnancy and early motherhood. The main contribution of this study is gaining a better understanding of the experiences of mothering young children while in prison. Unfortunately, the study suffers from several conceptual drawbacks that are presented below: | |
| **A.** | **Literature review** |  |
| 1. | There has been substantial research on mothers' experience of separation from their children while in prison (and, more broadly, trauma or stigmatized identity). It is only minimally reviewed here, but it would be helpful to include it. For example: Lovell, B. J., Steen, M. P., Brown, A. E., & Esterman, A. J. (2022). The voices of incarcerated women at the forefront of parenting program development: a trauma-informed approach to education. Health & Justice, 10(1), 1-16. | Thank you for your feedback. We have reviewed and included findings from the study you have suggested (Line no. 133; 154, 171). We have revised the introduction section to make it clearer. |
| 2. | More contexts needed to be added regarding the theoretical background of the study. This context would also lay the groundwork for the authors to provide more knowledge for the reader, which is necessary to understand the current study's findings. | This is similar to the suggestion made by reviewer 1 (addressed above) |
| 3. | Given the qualitative methodology and the cultural construction of motherhood, I was surprised to see no literature or framing with attention to the cultural and intersectional contexts. The only mentions of the Indigenous communities in the demographics and the findings despite that they represent 38% of the national female prison population. | Thank you for raising some interesting points. Exploring the cultural construction of motherhood would be an interesting area; however, this was not the focus of this study. We were interested in exploring needs of mothers in general. We did have a mix of both Indigenous and non-Indigenous mothers in our workshops, but data collection was undertaken in a group setting and it was not possible to distinguish how Indigenous mothers perceive motherhood compared to non-Indigenous mothers. It would be an interesting area for further research, which we have included as one of the recommendations of this study (Line no. 739-741) |
| 4. | Framing this study within its intersectional context and helping the readers understand what unique factors come into play as a result would increase the impact of this manuscript. This perspective can shed light on various systemic barriers found in the study. This could also help to understand the underlying mechanism of findings such as those mentioned on line 388: "Some mothers, predominantly those from remote Indigenous communities, perceived that prison policies and procedure discriminate and segregate mothers." See, for example. Potter, H. (2013). Intersectional criminology: Interrogating identity and power in criminological research and theory. Critical Criminology, 21(3), 305-318. Gueta, K. (2020). Exploring the promise of intersectionality for promoting justice-involved women's health research and policy. Health & Justice, 8(1), 1-10. |  |
| **B.** | **Results** |  |
| 5. | Most of the findings focus on negative and critical aspects of the mothers' experience. I was hoping to see more discussion of the interplay between facets of the experience. No direct references were made to the positive aspect of incarceration with children, particularly given the alternatives of separation from the child or losing custody. | We did include some positive experiences mentioned by mothers. For example, the mothers appreciated the antenatal program being provided in the prison, but they talked about some administrative barriers which need to be improved to improve their experience (Line no. 319 to 326). Other positive aspects of being with children have been mentioned in the results section. Despite most mothers acknowledging that having their young children with them in prison provides them with purpose, motivates them to change for better future, and improves their bond with their child, mothers wanted to see improvements in existing correctional policies so that they can exercise their motherhood independently (Line no. 374 – 379). Furthermore, they wanted to have smoother, more straight-forward processes to bring their child with them in prison (Line no. 383-391)  This study was conducted to provide input for developing a holistic program for imprisoned mothers, where mothers were asked about their experiences and needs as mothers. This prompted most women talked about what they want to change. It does not mean that all mothers were completely negative about existing correctional programs and policies. Most mothers appreciated existing programs, but they want some changes to improve their experiences, which has been discussed in the results section.  This study was conducted at the time when correctional centres resumed prison visits after several months of lockdown due to the coronavirus pandemic. Many mothers were not allowed to have their children with them in prison and in-person visits were suspended. This may have influenced a focus on negative aspects of rearing a young child while imprisoned. This has been included as one of the limitations of the study (Line no. 724-729) |
| 6. | Also, there is a need to explore further the variations in experiences described by mothers, with some experiencing no difficulties and others reporting extremely lengthy and complicated processes with futile results (line 382). One wonders whether the interview process and analysis were restrictive and not as open to the participants' perspectives as they might have been. Participants may have had concerns other than those of specific interest to the researchers, which may have been overlooked. Participants may experience both positive and negative experiences during incarceration. Thus, more information is needed on how participants integrated these experiences and overall evaluations of their experiences. | The findings presented in this paper were from workshops conducted with imprisoned mothers to explore their needs before coming to prison, in prison, and post release (this has been mentioned in the Methods section; Line no. 236 to 242). Mothers were not interviewed individually, therefore in-depth information about their experiences could not be explored (Line no. 739 – 746). We do acknowledge that there are some methodological limitations in the data collection tools employed in this study as this did not necessarily provide detailed accounts of mothers’ experiences (this has been mentioned as one of the limitations of the study; Line no. 745- 746). However, given the relative dearth of research in this area, using mothers’ voices to examine how they experience being pregnant and mothering a young child in prison is important. |
